# BRCA1 Promoter Hypermethylation in Malignant Breast Tumors and in the Histologically Normal Adjacent Tissues to the Tumors: Exploring Its Potential as a Biomarker and Its Clinical Significance in a Translational Approach

**DOI:** 10.3390/genes14091680

**Published:** 2023-08-25

**Authors:** Yassire Oubaddou, Mohamed Oukabli, Salma Fenniche, Abderrahim Elktaibi, Mohamed Reda Elochi, Abderrahmane Al Bouzidi, Zineb Qmichou, Nadia Dakka, Caroline Diorio, Antje Richter, Youssef Bakri, Rabii Ameziane El Hassani

**Affiliations:** 1 Laboratory of Biology of Human Pathologies (BioPatH), Faculty of Sciences, Mohammed V University in Rabat, Rabat 10001, Morocco; yassire.oubaddou@um5r.ac.ma (Y.O.); salma.fenniche@um5r.ac.ma (S.F.); n.dakka@um5r.ac.ma (N.D.); y.bakri@um5r.ac.ma (Y.B.); 2 Service of Anatomical Pathology, Military Hospital of Instruction Mohammed V (HMIMV-R), Faculty of Medicine and Pharmacy, Mohammed V University in Rabat, Rabat 10001, Morocco; oukablimohamed@yahoo.fr (M.O.); ab.elktaibi@gmail.com (A.E.); elochi2060@gmail.com (M.R.E.); 3 Cabinet Anatomie Pathologique Essaada, Rabat 10001, Morocco; albouzidi@gmail.com; 4 Medical Biotechnology Center, Moroccan Foundation for Advanced Science, Innovation and Research (MAScIR), Rabat 10001, Morocco; z.qmichou@mascir.ma; 5 Cancer Research Center, CHU de Québec—Université Laval Research Center, Oncology Division, Québec, QC G1R 3S3, Canada; caroline.diorio@crchudequebec.ulaval.ca; 6 Department of Social and Preventive Medicine, Faculty of Medicine, Université Laval, Québec, QC GIV 0A6, Canada; 7 Institute for Genetics, University Giessen, 35392 Giessen, Germany; antje.m.richter@gen.bio.uni-giessen.de

**Keywords:** BRCA1 promoter hypermethylation, breast cancers, normal adjacent tissue, benign breast lesions, methylation-specific PCR, biomarker

## Abstract

The hypermethylation status of the promoter region of the breast cancer 1 (*BRCA1)*, a well-known tumor suppressor gene, has been extensively investigated in the last two decades as a potential biomarker for breast cancer. In this retrospective study, we investigated the prevalence of *BRCA1* promoter methylation in 84 human breast tissues, and we correlated this epigenetic silencing with the clinical and histopathological parameters of breast cancer. We used methylation-specific PCR (MSP) to analyze BRCA1 promoter hypermethylation in 48 malignant breast tumors (MBTs), 15 normal adjacent tissues (NATs), and 21 benign breast lesions (BBLs). The results showed that *BRCA1* promoter hypermethylation was higher in MBTs (20/48; 41.67%) and NATs (7/15; 46.67%) compared to BBLs (4/21; 19.05%). The high percentage of BRCA1 hypermethylation in the histologically normal adjacent tissues to the tumors (NATs) suggests the involvement of this epigenetic silencing as a potential biomarker of the early genomic instability in NATs surrounding the tumors. The detection of BRCA1 promoter hypermethylation in BBLs reinforces this suggestion, knowing that a non-negligible rate of benign breast lesions was reported to evolve into cancer. Moreover, our results indicated that the *BRCA1* promoter hypermethylated group of MBTs exhibited higher rates of aggressive features, as indicated by the SBR III grade (14/19; 73.68%), elevated Ki67 levels (13/16; 81.25%), and Her2 receptor overexpression (5/20; 25%). Finally, we observed a concordance (60%) in *BRCA1* promoter hypermethylation status between malignant breast tumors and their paired histologically normal adjacent tissues. This study highlights the role of BRCA1 promoter hypermethylation as a potential useful biomarker of aggressiveness in MBTs and as an early marker of genomic instability in both histological NATs and BBLs.

## 1. Introduction

Breast cancer is one of the most commonly diagnosed cancers worldwide and a major cause of cancer-related deaths among women [1]. It is a complex and heterogeneous disease with a multifactorial etiology involving genetic, environmental, and lifestyle factors [2]. Mutations in the breast cancer 1 (BRCA1) gene, a well-known tumor suppressor gene, are associated with hereditary breast cancers, which constitute 5–10% of all breast cancer cases [3]. However, the majority of breast cancer cases are sporadic, with no family history of the disease, and the mechanisms underlying their development remain poorly understood [3].

Epigenetic alterations, including gene silencing through DNA hypermethylation, have emerged as crucial contributors to breast cancer development and progression [4]; BRCA1 promoter hypermethylation is the most studied epigenetic alteration in malignant breast tumors [5,6,7]. Interestingly, a study carried out on 237 triple-negative breast cancer (TNBC) cases indicated that the promoter hypermethylation of BRCA1 is more frequent than BRCA1 germline mutations, suggesting that both alterations can independently drive malignant breast tumorigenesis [8]. Indeed, this result is supported by the rarity of BRCA1 promoter hypermethylation in BRCA1 germline mutation carriers [9].

BRCA1 promoter hypermethylation correlates with decreased BRCA1 gene expression (epigenetic silencing), the loss of homologous recombination (HR) repair function [8,10], and, consequently, may promote genomic instability due to multiple BRCA1 gene-related functions in maintaining genome stability, such as DNA double-strand break repair, chromatin remodeling, stalled fork resolution, and R-loop resolution [11,12,13,14,15]. Therefore, BRCA1 DNA hypermethylation could be an early event accelerating breast tumorigenesis similarly to BRCA1 germline mutations [8]. In addition, BRCA1 promoter hypermethylation correlates with a higher risk of recurrence, reduced survival rates of breast cancer [16,17], and it can affect the response to certain chemotherapy agents and targeted therapies, such as platinum-derived therapy and PARP inhibitors [18,19]. Altogether, these results highlight the potential of exploring BRCA1 hypermethylation as a diagnostic and prognostic biomarker to improve the management of breast cancers, and further translational research is needed in this direction.

In this retrospective study, we investigated BRCA1 promoter hypermethylation in a cohort of 84 FFPE breast tissues (FFPE: formalin-fixed and paraffin-embedded) through the MSP technique (methylation-specific PCR), and we correlated it with different clinical and histopathological features of randomly selected malignant breast tumors. Our findings demonstrated a higher prevalence of BRCA1 promoter hypermethylation in malignant breast tumors (MBTs) and normal adjacent tissues (NATs) compared to benign breast lesion samples. Furthermore, we observed a concordance in the status of BRCA1 promoter hypermethylation between MBTs and their paired histologically normal adjacent tissues (‘NATs’). Interestingly, BRCA1 promoter hypermethylation was associated with the aggressive characteristics of breast cancer, including Scarff–Bloom–Richardson (SBR) grade III, elevated levels of ki67 expression, the overexpression of the oncogene human epidermal growth factor receptor (Her2), and the loss of estrogen and progesterone receptor expression. Our findings can contribute to a better understanding of the role of BRCA1 promoter hypermethylation in breast tumorigenesis, with potential clinical implications for the diagnosis, prognosis, and treatment of breast cancer. In addition, our results highlight that BRCA1 promoter hypermethylation could constitute an early event of genomic instability in the histologically normal breast tissues surrounding the tumors.

## 2. Materials and Methods

### 2.1. Study Subjects

This research received ethical approval from the Ethics Committee for Biomedical Research (CERB) of the Faculty of Medicine and Pharmacy in Rabat, with approval number 52/20.

We conducted a retrospective study using 84 breast tissue samples collected from patients diagnosed at the Department of Anatomical Pathology in the Military Hospital of Instruction Mohammed V in Rabat (HMIMV-R) from July 2017 to December 2019. Our cohort comprised 48 malignant breast tumors (MBTs), 15 of their normal adjacent tissues (NATs), and 21 benign breast lesions (BBLs). Those tissue samples were subjected to histological classification, following the guidelines of the World Health Organization (WHO) classification [20]. Furthermore, retrospective diagnosis confirmation was performed by experienced pathologists from the HMIMV-R. The selection of FFPE blocks was based on two criteria: (1) tumor cell sufficiency (more than 90% tumor cells in MBTs; absence of tumor cells in both NATs and BBLs) and (2) the availability of FFPE blocks. Normal adjacent tissues ‘NATs’ are defined as histologically normal tissues (absence of tumor cells) surrounding the malignant tissue. In this retrospective study, the distance between MBTs and NATs was not considered.

To collect clinicopathological data, we created a comprehensive database using patient medical files. Data not archived in patient medical files are not available and, therefore, are considered as missing data.

### 2.2. Cell Culture

The human MCF7 cell line was grown in DMEM (Dulbecco’s Modified Eagle’s Medium) with 10% SVF, 1% penicillin streptomycin, 1% glutamine, and 1% non-essential amino acids. The cell culture was performed in CO_2_ incubator (humidified environment set at 37 °C, with 5% CO_2_). Further, 300 × 10^3^ cells/well (6-well plates)/2 mL of complete medium were seeded, and genomic DNA was extracted on days 1, 2, 3, and 4 after seeding.

### 2.3. Genomic DNA Extraction from FFPE Tissues

The genomic DNA extraction was performed in 8 consecutive sections of 10 μm each, according to the manufacturer’s protocol ‘QIAamp DNA FFPE Tissue Kit (Qiagen)’, specially designed for purifying DNA from FFPE tissue sections [21,22]. The quality and concentration of the extracted genomic DNA were determined using the IMPLEN NanoPhotometer N60.

### 2.4. Genomic DNA Extraction from MCF7 Cell Line

Genomic DNA from human MCF7 cells was extracted using PureLink^®^ Genomic DNA Mini Kit (Invitrogen, K1820-01) according to the manufacturer’s protocol. The quality and concentration of the extracted genomic DNA were determined using the IMPLEN NanoPhotometer N60.

### 2.5. BRCA1 Promoter Hypermethylation Analysis

We analyzed the DNA hypermethylation status of the BRCA1 promoter gene using the methylation-specific PCR (MSP) technique. Since the MSP technique is not a quantitative approach, the MSP product detected during gel electrophoresis is qualified as methylated or hypermethylated.

Following genomic DNA extraction, we performed sodium bisulfite conversion using two kits: the Epitect Fast DNA Bisulfite Kit (Qiagen, 59826) and the EZ DNA Methylation kit (ZymoResearch, D5002), according to the manufacturer’s protocols [23]. We used 1000 ng of genomic DNA per sample for sodium bisulfite conversion.

To amplify the targeted BRCA1 promoter region, we used two pairs of primers ‘M: Hypermethylated and U: Unmethylated’ [24]. The U pair of primers was designed to amplify unmethylated DNA (sense: 5′-TTG GTT TTT GTG GTA ATG GAA AAG TGT-3’ and antisense: 5′-CAA AAA ATC TCA ACA AAC TCA CAC CA-3’). The M pair of primers was used to amplify hypermethylated DNA (sense: 5′-TCG TGG TAA CGG AAA AGC GC-3’ and antisense: 5′-AAA TCT CAA CGA ACT CAC GCC G-3’).

The HotStarTaq DNA Polymerase (Qiagen, Hilden, Germany) was used for PCR reaction according to the manufacturer’s protocol. The PCR parameters were: 95 °C for 15 min, followed by 40 cycles of 95 °C for 30 s, 56 °C for 30 s, and 72 °C for 45 s, and a final extension cycle of 72 °C for 10 min, all carried out in the Thermal Cycler (ProFlex PCR System; Thermo Fisher, Waltham, MA, USA).

The PCR products were separated through gel electrophoresis (MUPID-ONE 230 V; Deutscher) using a 2% agarose gel in tris-borate EDTA X1 buffer and visualized using ethidium bromide (BET). Finally, the hypermethylation status of the BRCA1 promoter region was determined by analyzing the gel images obtained with the ENDURO™ GDS Gel Documentation system (Labnet International Inc., Edison, NJ, USA).

### 2.6. Inclusion/Exclusion Criteria of MSP Results

These inclusion/exclusion criteria allow us to interpret our results with rigor, reliability, and credibility. Indeed, genomic DNA extracted from FFPE tissues is known to be extensively fragmented [25,26,27], and the sodium bisulfite conversion can further fragilize the genomic DNA. Therefore, the MSP results strongly depend on the quality of DNA extracted from FFPE tissues and on its conversion process. To optimize our experimentations, we used a specific kit for DNA extraction from FFPE tissues, known to improve the quality of extracted genomic DNA from FFPE tissues, and two credible sodium bisulfite conversion kits.

We explored 106 FFPE human breast tissues, and we included only the results obtained from 84 FFPE samples. All MSP results (22) showing any amplification with both primers (U and M) were excluded. Note that the majority of MSP results were obtained twice with both Epitect Fast DNA Bisulfite Kit (Qiagen, 59826) and the EZ DNA Methylation kit (ZymoResearch, D5002).

### 2.7. Statistical Analysis

Statistical analyses and graphical visualizations were performed using GraphPad Prism8. To assess the statistical significance of various associations, different tests were employed, including Fisher’s exact test, chi-square test, and unpaired *t*-test. To evaluate the concordance in BRCA1 promoter hypermethylation status between NATs and their matched MBTs, Cohen’s Kappa and McNemar’s tests were utilized. A *p*-value of less than 0.05 was considered statistically significant.

## 3. Results

### 3.1. Clinico-Histopathological Characteristics of Malignant Breast Tumor Patients

The median age of patients with malignant breast tumors (MBTs) was 50 years, with the youngest patient being 33 and the oldest being 70 years old (Table 1). Histologically, the majority of MBTs (41/48; 85.43%) were invasive carcinomas of no special type (NST). Additionally, 4 cases (4/48; 8.33%) were invasive medullary carcinoma, 1 case was metaplastic invasive carcinoma (1/48; 2.08%), 1 case was carcinoma ex pleomorphic adenoma of the breast (1/48; 2.08%), and 1 case was ductal in situ carcinoma with several invasive micro infiltrations (1/48; 2.08%).

We analyzed the aggressive feature data in malignant breast tumors and found that the in situ component was absent in half of the cases (21/42; 50%) (Table 1). Moreover, 37.5% (18/48) of cases displayed a tumor size greater than 3 cm, 46.15% (18/39) showed positive nodal status, and 29.17% (14/48) exhibited vascular emboli (Table 1). In addition, 55.32% (26/47) of cases were classified as SBR grade III and 64.86% (24/37) had a ki67 expression greater than or equal to 30% (Table 1). In addition, we observed that 66.67% (32/48) of cases tested negative for estrogen (ER) and progesterone (PR) receptors, and 12.50% (6/48) tested positive for Her2 receptor overexpression (Table 1). Regarding the 48 MBTs, 30 cases (30/48; 62.5%) were classified as triple-negative (ER^−^/Her2^−^), while 12 cases (12/48; 25%) were of the luminal Her2^−^ (ER^+^/Her2^−^) subtype. The remaining cases included 4 cases (4/48; 8.33%) of the luminal Her2^+^ (ER^+^/Her2^+^) subtype and 2 cases (2/48; 4.17%) of the Her2^+^ “only” (ER^−^/Her2^+^) subtype (Table 1).

### 3.2. BRCA1 Promoter Hypermethylation in Breast Tissue Samples

**Figure 1:**
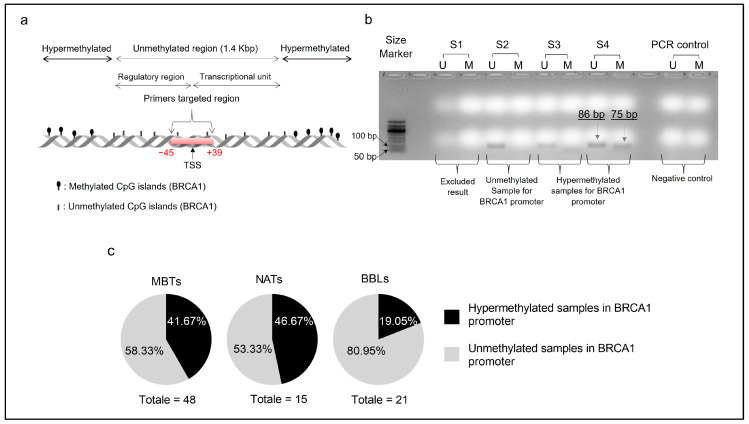
Results of methylation-specific PCR (MSP) analysis of the BRCA1 promoter in 84 FFPE samples. (**a**) Region targeted by primers for the MSP. The BRCA1 regulatory region is included in a large unmethylated sequence of 1.4 kilobase pairs (Kbp) flanked by two hypermethylated regions. (**b**) Electrophoresis gel image of MSP products for four malignant breast tumor samples (S1, 2,3, and 4). The PCR product “U” is 86 bp in length, while the PCR product “M” is 75 bp. (**c**) Portions of BRCA1 promoter hypermethylation and unmethylation in different breast tissue samples: Malignant Breast Tumors (48MBTs), Normal Adjacent Tissues (15NATs), and Benign Breast Lesions (21BBLs). MBTs: malignant breast tumors, NATs normal adjacent tissues to the tumor, BBLs: benign breast lesions, TSS: transcriptional start site, S: Sample, U: MSP product using pair of primers amplifying the unmethylated state of the targeted region. M: MSP product using the pair of primers M amplifying the hypermethylated state of the targeted region.

In this study, we examined the BRCA1 promoter hypermethylation status in 84 human breast tissues (48 MBTs, 15 NATs, and 21 BBLs) using methylation-specific PCR. According to Esteller et al. [24], the sense of primers U binds to the 1536 bp position of the Gen-Bank sequence “U37574”, while the sense of primers M binds to the 1543 bp position of the same sequence [24] (Figure 1a). The BRCA1 regulatory region is included in a large unmethylated sequence of 1.4 kilobase pairs (Kbp) flanked by two hypermethylated regions [28]. This regulatory region is selectively maintained in normal cells unmethylated to ensure BRCA1 gene expression [28]. The targeted region of the BRCA1 promoter is located approximately between positions −45 (1536 bp in “U37574”, sequence) and +39 (1619 bp in “U37574”, sequence), comprising the transcriptional start site, TSS (Figure 1a). The validation of primers was conducted in the MCF-7 cell line as a human breast cancer model of Luminal A subtype‘ER^+^, BRCA1^wt^ ’ [29]. Divers studies reported negative [30,31] or partial methylation of BRCA1in these cells [32,33], and our exploration reinforces the partial methylation of BRCA1 in MCF-7 cells (Figure S1). Interestingly, we observed a stable BRCA1 promoter methylation profile throughout the proliferation period of breast cancer MCF7 cells (4 days) (Figure S1). Our result highlights the stability of this epigenetic silencing in breast cancer cells independently of both the confluence state of cells and secreted cytokines in the extracellular medium over 4 days.

Figure 1b shows an example of MSP results of BRCA1. Sample 1 (S1) did not generate exploitable results and was, therefore, excluded from this study according to the inclusion/exclusion criteria of MSP results. Sample 2 (S2) is compatibilized as unmethylated for BRCA1, and both samples 3 and 4 (S3 and S4) are compatibilized as hypermethylated for the BRCA1 promoter (Figure 1b). Our study found BRCA1 promoter hypermethylation in 41.67% (20/48) of malignant breast tumors (MBTs) and 46.67% (7/15) of normal adjacent tissues (NATs) (Figure 1c and Table S1), strengthening a similar methylation profile between MBTs and NATs (Figure 1c and Table S1). Interestingly, we observed a non-negligible BRCA1 promoter hypermethylation of NATs compared to MBTs (Table 2). Regarding the 15 MBTs and their 15 paired NATs, 5 MBTs (33.33%) and 7 NATs (46.66%) showed BRCA1 promoter hypermethylation (Table 3). In contrast, only 19.05% (4/21) of benign breast lesions (BBLs) showed BRCA1 promoter hypermethylation (Figure 1c). Interestingly, of the 15 pairs of malignant breast tumors and their histologically normal adjacent tissues, 60% (3/5) exhibited concordance in their status of BRCA1 promoter hypermethylation, and 60% (6/10) exhibited concordance in their status of BRCA1 promoter unmethylation (Table 3).

### 3.3. Association between BRCA1 Promoter Hypermethylation and Aggressive Characteristics of Breast Cancer

**Figure 2:**
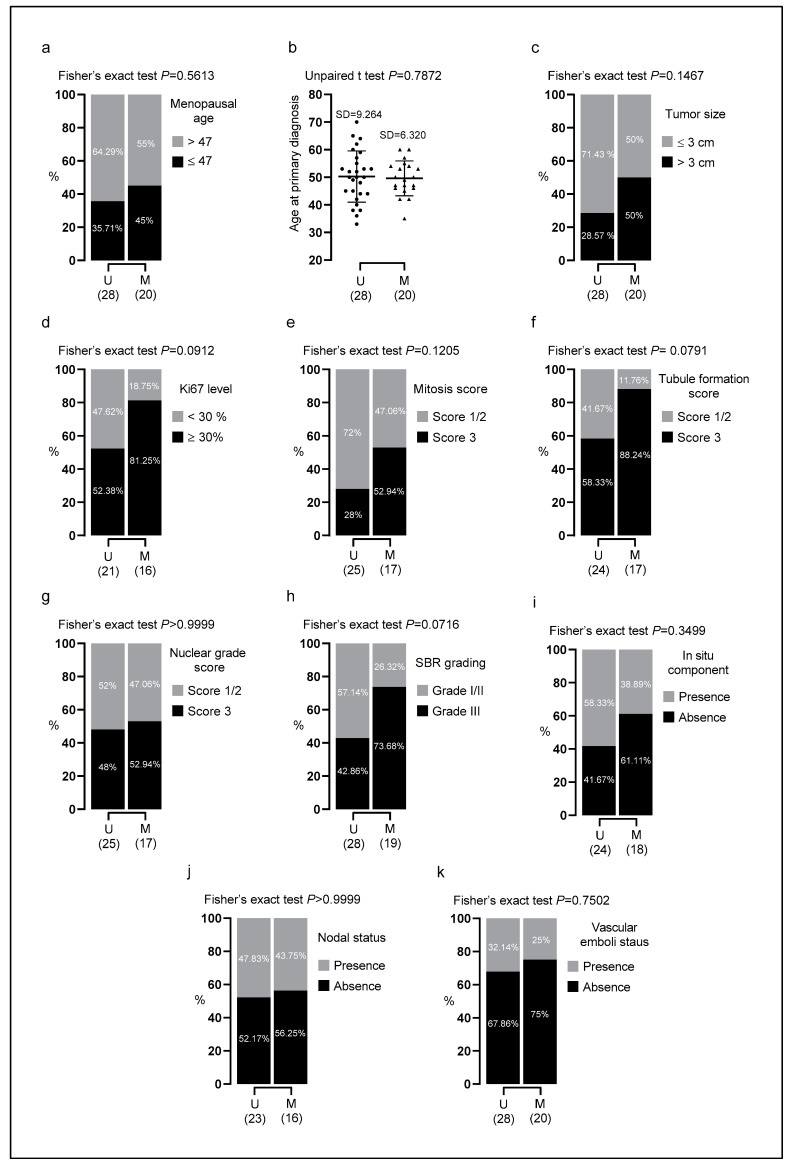
Association between BRCA1 promoter methylation and various clinical and histopathological characteristics of malignant breast tumors (*n* = 48): (**a**) menopausal age (≤47: premenopausal age; >47: postmenopausal age), (**b**) age at primary diagnosis, (**c**) tumor size, (**d**) Ki67 level, (**e**) mitosis score, (**f**) tubule formation score, (**g**) nuclear grade score, (**h**) SBR grading, (**i**) in situ component, (**j**) nodal status, (**k**) vascular emboli status. The letter “U” in each graph represents the group of BRCA1 unmethylated samples, while the letter “M” represents the group of BRCA1 hypermethylated samples. The missing data for each feature are presented in Table S2. The statistical significance was determined by GraphPad 8. SD: Standard Deviation; SRB: Scarff–Bloom–Richardson.

Analysis of BRCA1 promoter hypermethylation status using MSP revealed two distinct groups of MBTs: BRCA1 hypermethylated group (*n* = 20) and BRCA1 unmethylated group (*n* = 28) (Table S2). In our study, we observed a positive association between BRCA1 promoter hypermethylation and the aggressive features of breast cancer. Indeed, among the MBT cases with BRCA1 promoter hypermethylation, 45% (9/20) were diagnosed at age 47 years or younger, while in the BRCA1 unmethylated group, only 35.71% (10/28) fell within this age range at diagnosis (Figure 2a). Moreover, small tumor sizes (<3 cm) are more likely to be found in the unmethylated group (71.43% U vs. 28.57% M) compared to large tumor sizes (50% U vs. 50% M) (Figure 2c). Interestingly, we found that MBTs with BRCA1 promoter hypermethylation had increased levels (≥30%) of the cell cycle (G1, S, G2, and mitosis) biomarker Ki-67 (13/16; 81.25%) compared to the BRCA1 unmethylated group (11/21; 52.38%) (Figure 2d). In addition, we observed a higher frequency of mitosis score of 3 in the BRCA1 hypermethylated group (9/17; 52.94%) compared to the BRCA1 unmethylated group (7/25; 28%) (Figure 2e). These two latter findings suggest a potential association between BRCA1 promoter hypermethylation and the proliferative phenotype of breast cancer (Figure 2d,e). The presence of a tubule formation score of 3 was more frequent in the BRCA1 hypermethylated group (15/17; 88.24%) compared to the unmethylated group of MBTs (14/24; 58.33%) (Figure 2f). Regarding the nuclear grade feature, our analysis did not reveal any differences between the two groups (Figure 2g). The mitosis score, tubule formation score, and nuclear grade provide valuable parameters for breast cancer prognosis and aggressiveness determination using the SBR grading system [34]. Indeed, an SBR grade of III was found to be more prevalent in cases within the BRCA1 promoter hypermethylated group (14/19; 73.68%) compared to cases from the BRCA1 unmethylated group (12/28; 42.86%), suggesting an association between BRCA1 promoter hypermethylation and the aggressiveness of breast cancer (Figure 2h). In addition, the absence of in situ components was more commonly observed in BRCA1 hypermethylated MBTs (11/18; 61.11%) compared to BRCA1 unmethylated MBTs (10/24; 41.67%) (Figure 2i). Furthermore, our analysis revealed that BRCA1 promoter hypermethylation did not significantly impact the presence of vascular emboli or nodal invasion features (Figure 2j,k).

**Figure 3:**
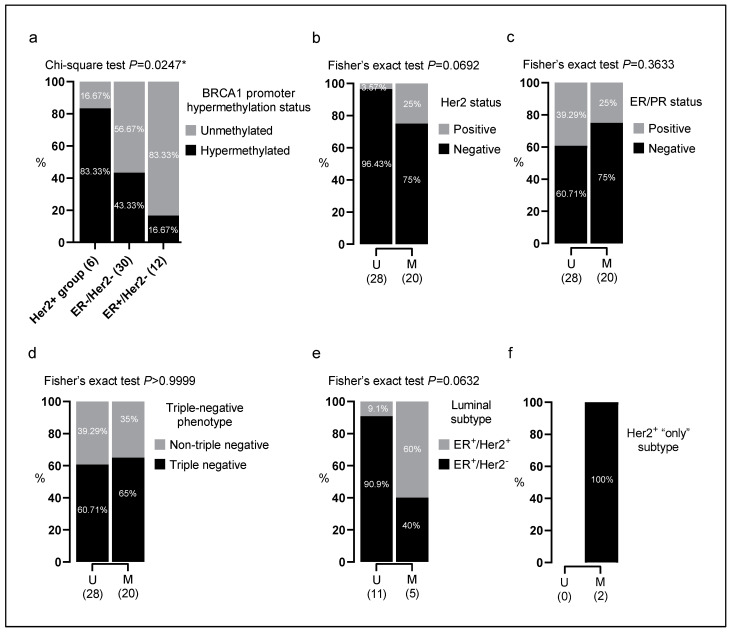
Association of BRCA1 promoter hypermethylation with Her2 overexpression and aggressive molecular subtypes of breast cancer: (**a**) molecular subtypes of breast cancer, (**b**) Her2 status, (**c**) ER/PR status, (**d**) triple-negative phenotype, (**e**) luminal subtype, (**f**) Her2+ “only” subtype. The letter “U” in each graph represents the group of BRCA1 unmethylated samples, while the letter “M” represents the group of BRCA1 hypermethylated samples. The missing data for each feature are presented in Table S2. The statistical significance was determined by GraphPad 8. * Statistical significance is affirmed by a *p*-value under 0.05. ER: Estrogen Receptor; PR: Progesterone Receptor; Her2: Human epidermal growth factor receptor 2.

Breast cancers that are Her2-positive are characterized by the overexpression of the Her2 protein [35]. These tumors were shown to carry unique molecular characteristics [36]. Regardless of their hormone receptors’ status, Her2-positive tumors often benefit from targeted therapies against the Her2 protein, such as trastuzumab [37]. Interestingly, Her2 overexpression was shown to have a strong prognostic value in breast cancer compared to hormone receptors (ER/PR) or nodal status [38]. Our findings indicate a pronounced prevalence of BRCA1 promoter hypermethylation in tumors with this oncogenic protein overexpression ‘*p* = 0.0247’ (Figure 3a). Furthermore, 43.33% (13/30) of the analyzed triple-negative breast tumors exhibited BRCA1 promoter hypermethylation (Figure 3a), strengthening the role of BRCA1 promoter hypermethylation in aggressive breast cancer subtypes.

Indeed, Her2 oncogene overexpression was more prevalent among cases with BRCA1 promoter hypermethylation (5/20; 25%) compared to the BRCA1 unmethylated group (1/28; 3.57%) (Figure 3b). Notably, Her2 receptor overexpression is known to be associated with more aggressive subtypes of breast cancer, including the luminal Her2^+^ (ER^+^/Her2^+^) and Her2^+^ “only” (ER^−^/Her2^+^) subtypes [39]. Consequently, the majority of cases (5/6: 83.33%) with luminal Her2^+^ and Her2^+^ “only” subtypes were found to be BRCA1 hypermethylated. Indeed, 100% (2/2) of Her2^+^ “only” cases were BRCA1 hypermethylated (Figure 3f), and 60% (3/5) of luminal Her2^+^ cases (ER^+^/Her2^+^) were BRCA1 hypermethylated (Figure 3e and Table S2), unlike the majority of the luminal Her2^−^ (ER^+^/Her2^−^) cases (10/12: 83.33), which were found BRCA1 unmethylated (Figure 3e and Table S2). More importantly, the loss of expression of estrogen (ER) and progesterone (PR) receptors was prevalent in cases with BRCA1 promoter hypermethylation (15/20; 75%) compared to the BRCA1 unmethylated group (17/28; 60.71%) (Figure 3c). Moreover, our analysis reveals a slight increase in BRCA1 promotor hypermethylation among the triple-negative (ER^−^/Her2^−^) subtype compared to the non-triple-negative subtype (Figure 3d).

## 4. Discussion

Breast cancer ‘BC’ is a real worldwide public health problem with an alarming incidence rate and mortality. The sporadic forms of BC, the most frequent ‘90–95%’, remain poorly characterized, and the epigenetic instability seems to play a pivotal role in sporadic BC compared to hereditary BC [40,41,42,43]. Exploring the diagnostic biomarkers for an early detection of this disease could improve its management, and during the last decade, interesting translational studies have evaluated the potential of BRCA1 promoter hypermethylation as a useful biomarker in BC.

This study used methylation-specific PCR (MSP) to investigate BRCA1 promoter methylation status in a cohort of 84 FFPE tissues by assessing its prevalence among different types of mammary tissues and its interpretation on different clinical and histopathological features of randomly selected malignant breast tumors. BRCA1 promoter methylation rates of 41.67%, 46.67%, and 19.05% were detected, respectively, in malignant breast tumors (MBTs), normal adjacent tissues (NATs), and benign breast lesions (BBLs). This significant BRCA1 promoter hypermethylation among samples from cancerous patients is in line with the report that BRCA1 promoter hypermethylation correlates positively with a high risk of breast cancer and aggressiveness [7]. Indeed, recently, the detection of BRCA1 promoter methylation has been extensively studied as a contributor to the development and progression of breast cancer [5,6,7,24].

The primers used in this study to detect BRCA1 promoter methylation have been shown in previous research to correlate with reduced expression of the BRCA1 gene [16]. This effect has also been observed in malignant breast tumors with BRCA1 germline mutations, a genetic alteration that was found to promote genome instability in normal human mammary cells [8,11,12,13].

The BRCA1 promoter methylation rate of MBTs (41.67%) is in concordance with other studies using the same primers, which reported rates ranging from 27% to 59% [44,45]. Interestingly, certain histopathological features, such as ER/PR negative status, were found to be associated with BRCA1 aberrant DNA methylation [7]. Consequently, the enrichment of MBTs subset by tumors harboring an ER/PR negative status (66.67%) may contribute to enhancing the observed BRCA1 promoter methylation rate (Table 1). Indeed, BRCA1 promoter methylation, in our study, was more pronounced among ER/PR-negative MBTs than ER/PR-positive MBTs but without pointing to statistical significance (*p* = 0.3633) (Figure 3c).

Previous studies have suggested that BRCA1 promoter methylation and BRCA1 germline mutations are more commonly observed in triple-negative breast tumors compared to non-triple-negative breast tumors [7,46,47]. However, our results did not reflect this finding (Figure 3d), which may be due to the heterogeneity in the group of patients with an unmethylated BRCA1 promoter, which could contain potential cases with BRCA1 germline mutations. We can apply the same reasoning to question the association between the other aggressive features of the MBT group and BRCA1 promoter methylation reported in this study, especially since there are shared clinical and molecular features (BRCAness) between breast tumors associated with BRCA1 mutations and those with BRCA1 promoter methylation, as previously noted [8]. Indeed, the distribution of the analyzed MBTs according to the age at primary diagnosis revealed that the methylated group of MBTs formed a more homogeneous group of tumors compared to the unmethylated group, which exhibited greater diversity by including cases from a wider age range (standard deviation (SD): 6.320 (M) vs. 9.264 (U)) (Figure 2b). Nevertheless, we noticed higher incidences of aggressive characteristics in the methylated group. These included a higher frequency of SBR grade III tumors, elevated levels of ki67 (≥30%), larger tumor size (>3 cm), absence of in situ component, negative ER/PR (estrogen and progesterone) receptor status, and positive Her2 receptor overexpression.

Regarding the benign breast lesions (BBLs), we reported 19.05% of BRCA1 promoter hypermethylation in the current study compared to MBTs (41.67%), and the high frequency of BRCA1 methylation in MBTs compared to BBLs is consistent with a report by Payadar et al. [48]. This aberrant DNA methylation of BRCA1 in non-cancerous mammary tissues needs a deep investigation in the aim to illustrate its early involvement in cell transformation. Some BBLs are reported to progress in breast cancer [49,50,51], and the proliferative character of BBLs seems to increase their risk for developing breast cancer [50]. However, the molecular mechanisms influencing this progression are still unknown. The hypermethylation of the BRCA1 promoter in BBLs could be considered as an early molecular biomarker for both genetic and epigenetic instabilities, and, consequently, patients with BBLs harboring BRCA1 hypermethylation can be considered patients at risk. Indeed, similar promoter hypermethylation is reported in 54 MBTs and 10 BBLs, suggesting that this epigenetic deregulation could be an early event in breast carcinogenesis [52].

The epigenetic silencing of a tumor suppressor gene in normal (non-cancerous) breast tissue adjacent to tumor lesions may render it a putative site of recurrent breast neoplastic transformation [53]. In the current study, a high rate of BRCA1 promoter methylation (46.67%) was observed in normal adjacent tissue samples. A previous analysis of single cells showed the presence of BRCA1 promoter methylation in normal mammary epithelial cells belonging to the histologically normal mammary adjacent tissues [54]. This disputes the risk of false-positive MSP results that may be caused by contamination by cancer cells when analyzing histologically normal adjacent tissues using the MSP technique; hence, any detected methylation in this type of tissue refers to the BRCA1 promoter methylation of normal cells. The rate of BRCA1 promoter methylation in NATs has been reported to vary depending on the distance between the normal tissue site and the tumor [55,56]. Specifically, the use of normal adjacent tissues 3 to 5 cm far from tumors showed a significant rate of BRCA1 promoter methylation, 51.90% [56], while distant normal tissues show no detection of BRCA1 promoter methylation [55].

In the current study, 60% of the MBTs and their paired NAT showed concordant BRCA1 promoter methylation status (Kappa = 0.182, *p* = 0.6831) (Table 3). The frequent detection of concordant aberrant DNA methylation in histologically normal adjacent tissues and tumor tissues has been attributed to various theories, including “field of cancerization”, “microenvironmental factors”, “BRCA1 constitutional methylation”, and “age-related DNA methylation changes” [15,53,57]. According to the “field of cancerization” theory, preexisting fields of normal cells with molecular alterations (e.g., DNA methylation) may drive tumorigenesis, and some of those alterations may persist in the developed tumors [53]. Previous reports have shown that normal breast adjacent tissues carry tens of thousands of epigenetic changes, with 30% of them being shared with their matched tumors [58]. Moreover, constitutional BRCA1 promoter methylation was detected in 4–7% of newborns and young females [59], which may constitute an early driver of breast tumorigenesis. Notably, aberrant BRCA1 DNA methylation detected in the blood of females increases the risk of triple-negative breast tumors [15].

In addition to its potential diagnostic biomarker role, methylated BRCA1 could be epigenetically reactivated. One possible therapeutic approach could be epigenetic editing using CRISPR-based techniques, as it offers the potential for precision therapy and personalized medicine. This approach could enable targeted reactivation of BRCA1, specifically in tumor cells, potentially restoring its tumor-suppressive function and inhibiting cancer progression [60,61,62]. However, it is important to note that epigenetic editing technologies are still in the early stages of development, and their clinical application for cancer treatment is an active area of research. Further studies and advancements in this field are needed to fully explore the potential of epigenetic editing as a therapeutic strategy for BRCA1-related cancers.

## 5. Conclusions

To summarize, our study shows that BRCA1 promoter methylation is a common alteration in malignant breast tumors and their adjacent normal tissues. Furthermore, tumors with this epigenetic mark were more likely to exhibit undifferentiated (score 3 of tubule formation) and hyperproliferative (score 3 of mitosis and high levels of Ki67%) phenotypes, lack of estrogen and progesterone receptors, overexpression of Her2 oncogene, and absence of in situ components. The high concordance in methylation status between tumor and normal tissues suggests that there is a consistent regulatory pathway driving BRCA1 epigenetic silencing in both types of tissues. These findings are particularly relevant given the increasing incidence of breast cancer in low- and middle-income countries (LMICs), where access to early detection and treatment options is limited, and the majority of breast-cancer-related deaths occur. The MSP technique used in this study is known for its low cost and convenience, as well as its high sensitivity to CpG methylation detection. Therefore, validating BRCA1 promoter methylation detection using this technique as a diagnostic and prognostic biomarker has the potential to improve early detection and treatment options, ultimately reducing the morbidity and mortality associated with breast cancer in LMICs.

Our results, supported by the literature, propose BRCA1 promoter hypermethylation as a potential useful biomarker for breast tumor aggressiveness, as well as an early biomarker for both BBLs at risk to progressing into cancer and NATs harboring the tumors at risk of recurrence. Normal adjacent tissues, usually used as normal control, are histologically non-cancerous but present a deep deregulation in proinflammatory pathways and genetic/epigenetic instabilities. Further translational studies exploring the relationship between BRCA1 promoter hypermethylation and the loss/decrease in its function in terms of DNA damage in both NATs and BBLs could reinforce the potential role of BRCA1 methylation as an early biomarker for patients at risk of developing cancer, as well as the clinical application of BRCA1 promoter methylation in the management of cancerous and non-cancerous breast diseases.

## Figures and Tables

**Table 1:** Clinical and histopathological characteristics of the study population.

Characteristics		MBTs (*n* = 48)		NATs (*n* = 15)		BBLs (*n* = 21)	
		*n*	%	*n*	%	*n*	%
Age	Median age	50 years		48 years		22 years	
	≤47 years	19	39.58	7	46.67	19	90.48
	>47 years	29	60.42	8	53.33	2	9.52
Tumor size	≤3 cm	30	62.50	-	-	-	-
	>3 cm	18	37.50	-	-	-	-
Tubule formation score	Score 1/2	12	29.27	-	-	-	-
	Score 3	29	70.73	-	-	-	-
	Missing data	7					
Nuclear grade score	Score 1/2	21	50	-	-	-	-
	Score 3	21	50	-	-	-	-
	Missing data	6					
Mitosis score	Score 1/2	26	61.90	-	-	-	-
	Score 3	16	38.10	-	-	-	-
	Missing data	6					
SBR grading	Grade I/II	21	44.68	-	-	-	-
	Grade III	26	55.32	-	-	-	-
	Missing data	1					
Ki67 level	<30%	13	35.14	-	-	-	-
	≥30%	24	64.86	-	-	-	-
	Missing data	11					
In situ component	Absence	21	50	-	-	-	-
	Presence	21	50	-	-	-	-
	Missing data	6					
Vascular emboli	Absence	34	70.83	-	-	-	-
	Presence	14	29.17	-	-	-	-
Nodal status	Negative	21	53.85	-	-	-	-
	Positive	18	46.15	-	-	-	-
	Missing data	9					
ER/PR status	Negative	32	66.67	-	-	-	-
	Positive	16	33.33	-	-	-	-
Her2 status	Negative	42	87.50	-	-	-	-
	Positive	6	12.50	-	-	-	-
Molecular subtype	ER^+^/Her2^−^	12	25.00	-	-	-	-
	ER^+^/Her2^+^	4	8.33	-	-	-	-
	ER^−^/Her2^+^	2	4.17	-	-	-	-
	ER^−^/Her2^−^	30	62.50	-	-	-	-

MBTs: Malignant Breast Tumors; NATs: Normal Adjacent Tissues; BBLs: Benign Breast Lesions; SRB: Scarff–Bloom–Richardson; ER: Estrogen Receptor; PR: Progesterone Receptor; Her2: Human epidermal growth factor receptor 2. -: Data not analyzed (NATs) or not available (BBLs).

**Table 2:** BRCA1 promoter hypermethylation: differences between MBTs, NATs, and BBLs.

Group vs. Group	*p*-Value	Statistical Tests
MBTs vs. NATs	0.7718	Fisher’s exact test
MBTs vs. BBLs	0.0999	Fisher’s exact test
NATs vs. BBLs	0.1410	Fisher’s exact test

MBTs: Malignant Breast Tumors; NATs: Normal Adjacent Tissues; BBLs: Benign Breast Lesions.

**Table 3:** Concordance in BRCA1 promoter hypermethylation (M) and unmethylation (U) status between 15 pairs of malignant breast tumors (MBTs) and their matched normal breast adjacent tissues (NATs).

		MBTs		McNemar’s Test	Cohens Test
		U	M		
NATs	U	6	2	*p* = 0.6831	Kappa = 0.182
	M	4	3		

MBTs: Malignant Breast Tumors; NATs: Normal Adjacent Tissues; U: Unmethylated; M: Hypermethylated.

## Data Availability

Archived datasets are not available due to ethical restrictions.

## Supplementary Materials

The following supporting information can be downloaded at: https://www.mdpi.com/article/10.3390/genes14091680/s1, Table S1: BRCA1 promoter methylation: portions in MBTs, NATs, and BBLs. Table S2: BRCA1 promoter methylation status and its association with clinico-histopathological features in 48 malignant breast tumors. Figure S1: Gel electrophoresis image representing the hypermethylation analysis of BRCA1 promoter via MSP.
