# Supplementary material for: BRCA1 Promoter Hypermethylation in Malignant Breast Tumors and in the Histologically Normal Adjacent Tissues to the Tumors: Exploring Its Potential as a Biomarker and Its Clinical Significance in a Translational Approach
Source: Genes (Basel). 2023 Aug 25;14(9):1680. doi: 10.3390/genes14091680 (PMC10530732; doi:10.3390/genes14091680)
Supplement: Supplementary file 1 [file genes-14-01680-s001.zip › genes-2556484-supplementary.pdf]

Table S1: BRCA1 promoter methylation: portions in MBTs, NATs, and BBLs.

-	*n*	Portions of BRCA1 promoter methylation	
MBTs	48	20/48	41.67%
NATs	15	7/15	46.67%
BBLs	21	4/21	19.05%

MBTs: Malignant Breast Tumors; NATs: Normal Adjacent Tissues; BBLs: Benign Breast Lesions.

Table S2: BRCA1 promoter methylation status and its association with clinico-histopathological features in 48 Malignant Breast Tumors.

Total of cases = 48		n	Unmethylated n=28 (58.33%)	%	Methylated n=20 (41.67%)	%	Missing data
Age	≤ 47	19	10	35.71 (10/28)	9	45 (9/20)	-
	> 47	29	18	64.29 (18/28)	11	55 (11/20)	
Tumor size	≤3 cm	30	20	71.43 (20/28)	10	50 (10/20)	-
	>3 cm	18	8	28.57 (8/28)	10	50 (10/20)	
Tubule formation score	Score 1/2	12	10	41.67 (10/24)	2	11.76 (2/17)	7
	Score 3	29	14	58.33 (14/24)	15	88.24 (15/17)	
Nuclear grade score	Score 1/2	21	13	52 (13/25)	8	47.06 (8/17)	6
	Score 3	21	12	48 (12/25)	9	52.94 (9/17)	
Mitosis score	Score 1/2	26	18	72 (18/25)	8	47.06 (8/17)	6
	Score 3	16	7	28 (7/25)	9	52.94 (9/17)	
SBR grading	Grade I/II	21	16	57.14 (16/28)	5	26.32 (5/19)	1
	Grade III	26	12	42.86 (12/28)	14	73.68 (14/19)	
Ki67 level	<30 %	13	10	47.62 (10/21)	3	18.75 (3/16)	11
	≥ 30 %	24	11	52.38 (11/21)	13	81.25 (13/16)	
In situ componen t	Absence	21	10	41.67 (10/24)	11	61.11 (11/18)	6
	Presence	21	14	58.33 (14/24)	7	38.89 (7/18)	
Vascular emboli	Absence	34	19	67.86 (19/28)	15	75.00 (15/20)	-
	Presence	14	9	32.14 (9/28)	5	25.00 (5/20)	
Nodal status	Negative	21	12	52.17 (12/23)	9	56.25 (9/16)	9
	Positive	18	11	47.83 (11/23)	7	43.75 (7/16)	
ER/PR status	Negative	32	17	60.71 (17/28)	15	75 (15/20)	-
	Positive	16	11	39.29 (11/28)	5	25 (5/20)	
Her2 status	Negative	42	27	96.43 (27/28)	15	75 (15/20)	-
	Positive	6	1	3.57 (1/28)	5	25 (5/20)	
Molecular subtypes	ER+/PR+/Her2-	12	10	35.71 (10/28)	2	10 (2/20)	-
	ER+/PR+/Her2+	4	1	3.57 (1/28)	3	15 (3/20)	
	ER-/PR-/Her2+	2	0	0.00	2	10 (2/20)	
	ER-/PR-/Her2-	30	17	60.71 (17/28)	13	65 (13/20)	

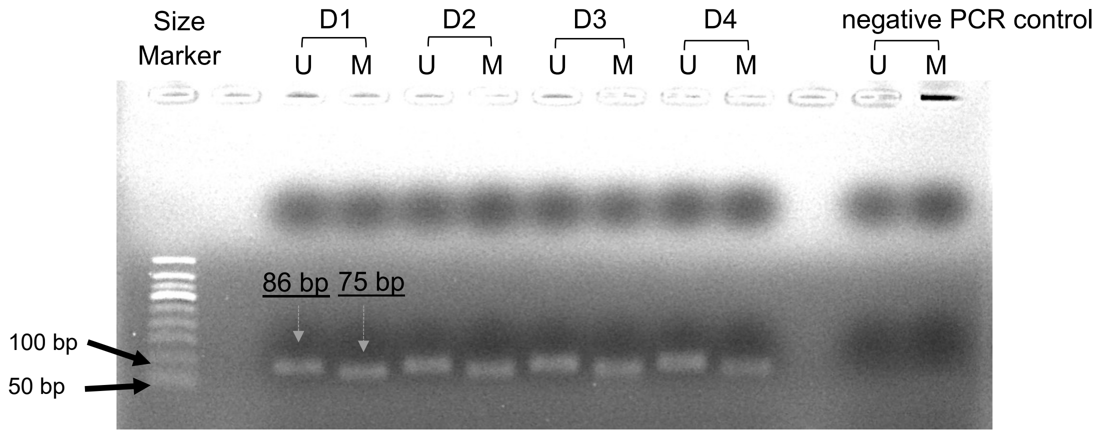

Figure S1: Gel electrophoresis image representing the hypermethylation Analysis of BRCA1 promoter via MSP. This analysis was conducted using MCF7 cultured cells, seeded at an initial concentration of 300 x 10^3 cells per well (plates of six wells). Genomic DNA was extracted at D1, D2, D3 and D4 (D:Day). U: MSP product using pair of primers amplifying the unmethylated state of the targeted region. M: MSP product using the pair of primers M amplifying the hypermethylated state of the targeted region.
